# Impact of Oral Microbiome in Periodontal Health and Periodontitis: A Critical Review on Prevention and Treatment

**DOI:** 10.3390/ijms23095142

**Published:** 2022-05-05

**Authors:** Mattia Di Stefano, Alessandro Polizzi, Simona Santonocito, Alessandra Romano, Teresa Lombardi, Gaetano Isola

**Affiliations:** 1 Department of General Surgery and Surgical-Medical Specialties, School of Dentistry, University of Catania, 95124 Catania, Italy; mattiadistefano@live.it (M.D.S.); gaetano.isola@unict.it (G.I.); 2 Department of General Surgery and Surgical-Medical Specialties, Unit of Hematology, University of Catania, 95124 Catania, Italy; alessandra.romano@unict.it; 3 Department of Health Sciences, Magna Græcia University, 88100 Catanzaro, Italy; drteresalombardi@libero.it

**Keywords:** oral microbiome, periodontal disease, periodontitis, periodontal treatment, dysbiosis, oral disease, oral health, Microbiome, Periodontal Health, Periodontal defects

## Abstract

The skin, oral cavity, digestive and reproductive tracts of the human body harbor symbiotic and commensal microorganisms living harmoniously with the host. The oral cavity houses one of the most heterogeneous microbial communities found in the human organism, ranking second in terms of species diversity and complexity only to the gastrointestinal microbiota and including bacteria, archaea, fungi, and viruses. The accumulation of microbial plaque in the oral cavity may lead, in susceptible individuals, to a complex host-mediated inflammatory and immune response representing the primary etiological factor of periodontal damage that occurs in periodontitis. Periodontal disease is a chronic inflammatory condition affecting about 20–50% of people worldwide and manifesting clinically through the detection of gingival inflammation, clinical attachment loss (CAL), radiographic assessed resorption of alveolar bone, periodontal pockets, gingival bleeding upon probing, teeth mobility and their potential loss in advanced stages. This review will evaluate the changes characterizing the oral microbiota in healthy periodontal tissues and those affected by periodontal disease through the evidence present in the literature. An important focus will be placed on the immediate and future impact of these changes on the modulation of the dysbiotic oral microbiome and clinical management of periodontal disease.

## 1. Introduction

The skin, oral cavity, digestive and reproductive tracts of the human body are home to cells of symbiotic and commensal microorganisms, which account for over 90% of the total human cellular makeup. Those microorganisms are organized in complex ecological communities whose composition may be significantly variable both within and among individuals depending on the age, lifestyle, and genetics of the host [1,2,3].

Periodontitis is a common chronic inflammatory condition that, if not adequately treated, may lead to the gradual destruction of the structural components of the teeth-supporting apparatus (cementum, periodontal ligament, alveolar bone, and gingival tissue) [4]. Periodontal disease represents a public health problem, affecting about 20–50% of people worldwide, and its global burden is predicted to increase in the future, above all due to the general aging population [5]. Gingival inflammation, clinical attachment loss (CAL), radiographic assessed resorption of alveolar bone, presence of periodontal pockets, gingival bleeding upon probing, and teeth mobility are all specific clinical signs of periodontitis that, may lead to premature teeth loss in advanced stages [4].

The primary etiological factor of periodontal damage is the host-mediated inflammatory and immune responses to the accumulation of microbial plaque and its diffusible enzymes, such as lipases, proteases, and nucleases [6] along with important individual causal factors, such as genetic and epigenetic susceptibility (i.e., single nucleotide polymorphism) [7], lifestyle factors (i.e., a diet low in vitamin C and D and other micronutrients [8,9], tobacco-using [10,11]) and various systemic diseases, like osteoporosis, atherosclerosis or diabetes, that may exacerbate the onset and the progression of periodontal disease [12].

Considering the growing global prevalence of periodontal disease and its potential irreversible damage to soft and connective teeth-supporting tissues, it is crucial to foster a culture of oral prevention through the promotion of adequate oral hygiene, abstention, and/or drastic reduction of tobacco use, excessive alcohol consumption, and exposition to stressing stimuli [13,14] as well as the elaboration of periodontal non-surgical treatment protocols effective in reducing or eliminating the pathogenicity of oral biofilm.

The purpose of this review is to provide recent literature updates regarding oral microbiome in periodontal health and disease focusing on oral microbiota maturation during life, its characteristics in health and its shifting in periodontitis and the impact of environmental factors and periodontal therapy.

## 2. Microbiome in the Oral Cavity

The hard surfaces of teeth and the soft tissues of the oral mucosa are colonized by one of the most heterogeneous microbial communities found in the human organism, ranking second in terms of species diversity and complexity only to the gastrointestinal microbiota and including bacteria, archaea, fungi, and viruses [15,16].

This complexity is due to the distinct environmental conditions in the different sites of the oral cavity (teeth, gingival sulcus, attached gingiva, tongue, non-keratinized cheek mucosa, lip, and hard and soft palate) that provide several different habitats for specific microbial colonization and growth. In these micro-environments, microorganisms can find the ideal condition to form highly structurally and functionally organized surface-associated communities (biofilms) immersed in an extracellular polymeric matrix (EPM) [17]. The EPM of mature biofilm consists of an ensemble of extracellular polymeric substances (water, polysaccharides, proteins, lipids, and DNA) that promote microorganisms’ colonization and stabilize microbial communities. Furthermore, the EPM maintains the biofilm tightly associated with the host tissues, facilitates inter- and/or intra-species interactions, and provides protection against host defense and drugs [18].

Bacteria are currently the most well-studied inhabitants of the oral cavity. Microbial components of the oral bacteriome do not exist as individual cells but perform microbial interactions to coordinate their activities. The oral microbial interactions mainly include synergic metabolic interactions or competition for nutrients, horizontal gene transfer, interference in signaling mechanisms to acquire a competitive advantage during colonization, and competition with other microbial organisms [19].

Gram-positive and Gram-negative oral bacteria in biofilms can modify their phenotype in response to cell density through a cell-cell signaling system known as *quorum sensing* (QS). It involves small diffusible signal molecules that bacteria synthesize and secrete to coordinate a variety of their activities—including biofilm formation and growth, adaptation to changes in the oral environment, the acquisition of a competitive advantage against potential competitors, and the expression of virulence factors that allow pathogens to cause disease [20]. This intercellular communication system was first studied and described in *Vibrio Fischer*, a marine organism able to produce light in response to an autoinducer through the proteins LuxI and LuxR. This LuxI-LuxR family proteins-relied system has been identified, with variable pathways, in many Gram-negative bacteria. It is related to both physiological and pathological bacterial functions, ranging from bioluminescence and plasmid conjugal transfer to the swarming motility and the synthesis of virulence-related factors [21].

The emergence and the development of cultural-independent techniques, such as real-time polymerase chain reaction (PCR)-based methods, highly conserved 16S ribosomal-RNA (rRNA) gene sequencing, and shotgun metagenomic libraries [22] along with next-generation DNA sequencing (NGS) methods, has given further evidence of the heterogeneous oral microbiome composition and has highlighted the contribution of this polymicrobial community in maintaining health or determining the onset of oral and systemic diseases [16,23,24,25]. For example, a study analyzing the human oral microbial composition in healthy subjects and subjects affected by periodontal diseases, using PCR-amplification, purification, cloning, and sequencing of 16S rRNA genes, estimated the presence of 415 species in the subgingival plaque (counting about 500 species if the other oral surfaces are considered) the presence of over 500 bacterial species, with about 60% of clones belonging to recognized species and the residual 40% being novel phylotypes [22]. However recent evidence showed that the results of oral microbiota biodiversity profiling are influenced by both DNA extraction strategies and targeted 16S rRNA hypervariable regions [26].

**Figure 1:**
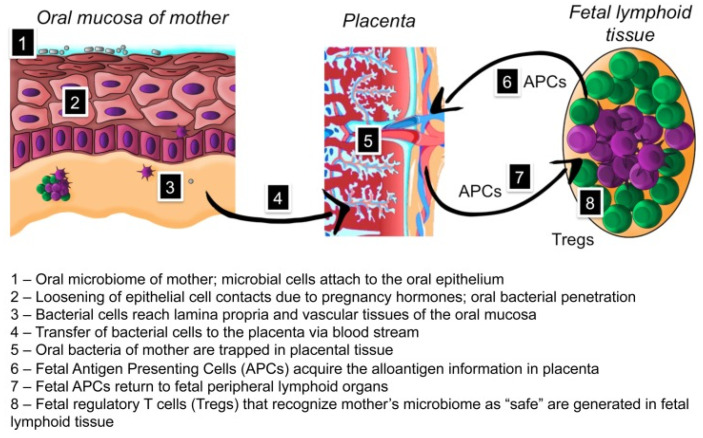
The hypothesis proposed by [27] suggests that the acquisition of antigen information by the Fetal Antigen Presenting Cells (APCs) through the placental tissue during pregnancy inducesthe development of fetal tolerance toward the oral microbiome of the mother and the consequent safe acquisition of a normal microbiome by the newborn. Under the terms of the Creative Commons Attribution License (CC BY).

The acquisition of the oral microbiome takes place in a dynamic process that involves the interaction between host genetics, the host immune system, and exposure to local and external environmental factors. During the prenatal period, a crosstalk between the maternal microbial antigens and the fetal antigen-presenting cells (APCs) through placental tissues (Figure 1) induces prenatal tolerance to the mother microbiome, allowing a safe acquisition of a normal microbiome [27,28]. Eutocic or dystocic delivery mode determinates the type of microorganisms that a child is first exposed to; vaginally delivered infants have shown to own a microbiome dominated by characteristic vaginal canal bacterial species, such as *Lactobacillus*, *Prevotella*, or *Sneathia*, while in infants born by Caesarean section skin surface-associated specieshas been found—predominantly *Staphylococcus*, *Corynebacterium*, and *Propionibacterium* spp. [29]. 3-month-old breast-fed infants show higher colonization with oral lactobacilli than formula-fed infants, suggesting the feeding method also influences the newborn microbial composition [30]. In addition to the maternal vertical transmission mechanism, a later horizontal transmission mechanism plays a key role in acquiring the microbiome since the newborn is exposed to other potential colonization sources [30]. A few minutes after birth, newborn bacteriomes in the oral cavity, nasopharynx, intestine, and skin are characterized by a homogeneous composition [31].

A remarkable event for increasing microbial diversity is teeth eruption since it provides new adhesion surfaces for microbial colonization. By the age of three, the oral microbiome is already complex and includes six bacterial phyla: *Firmicutes*, *Proteobacteria*, *Actinobacteria*, *Bacteroidetes*, *Fusobacteria*, and *Spirochaetes* with a prevalence of *Proteobacteria*, in particular *Gammaproteobacteria* class (*Pseudomonaceae*, *Moraxellaceae*, *Pastereullaceae*, *Enterobacteriaceae* families) [32]. The exfoliation of deciduous teeth for the replacement of the primary teeth considerably modifies the oral microbial habitat and leads toan increased proportion of the *Prevotellaceae* family (mainly genus *Prevotella*), *Veillonellaceae* family, *Spirochaetes*, and candidate division TM7 [32]. The oral microbiomes of healthy adult individuals show a similar composition at the genus level, with a relative abundance of 11 genera included in the phyla *Actinobacteria*, *Fusobacteria*, *Proteobacteria*, *Firmicutes*, and *Bacteroidetes* and considerable variations in species and strains, mainly related to demographic, anthropometric and environmental factors [33].

## 3. Oral Microbiome in Health and Its Shifting in Periodontitis

Oral bacteria in oral cavity are organized in structures known as “biofilm”, that is a complex protective structure in which bacterial communities are immersed in an extracellular matrix giving protection and resistance to the penetration of external agents [34]. Once established, the oral microbiome residing in the oral cavity co-evolves with the host and is maintained by a bidirectional interaction between the microbiome and the host, with the host and its microbes coexisting harmoniously. This interaction involves host- and microbe-derived factors contributing to the differentiation and maturation of the host mucosa, the development of the host immune system, and the prevention of invasion and growth of foreign and potentially harmful microorganisms (colonization resistance) [33]. For instance, some bacterial species of the oral cavity, such as certain strains of *S. mutans*, can prevent the invasion and the possible growth of endogenous microbial agents by synthesizing small antimicrobial peptides known as bacteriocins in a process regulated by quorum-sensing molecules [35].

Saliva, with its organic and inorganic constituents, plays a crucial role in maintaining oral health and regulating the healthy oral microbiota:

1. providing an acquired enamel and mucosal pellicle that represent the basis for the initial colonization of hard and soft tissues by the microorganisms;
2. diluting and eliminating microorganisms and dietary components, like carbohydrates and acids, from the mouth;
3. maintaining, in tolerant individuals, the microbial ecosystem through the antimicrobial action of certain proteins, such as lysozyme, proline-rich proteins, peroxidase, histatins, lactoferrin, and immunoglobulins, in particular secretory IgA (sIgA) and IgM;
4. maintaining a physiological constant pH (6.5–7) through different buffer systems; [36].

The oral microbiome of healthy individuals shows a bacterial prevalence of members of the phyla *Firmicutes*, *Proteobacteria*, *Actinobacteria*, *Bacteroidetes*, *Fusobacteria,* and *Spirochaetes* and also a less abundant fungal core, including microbes of the genera *Candida*, *Cryptococcus*, *Fusarium*, *Aspergillus,* and others [37]. A study analyzing the diversity in the composition of the bacterial oral community in ten healthy individuals shows that 15 bacterial genera were conserved among all ten of them, with significant interindividual differences at the species and strain level [38]. Mounting evidence that also fungi, archaea and sometimes viruses and/or other parasites inhabit the oral cavity implies that inter-kingdom microbial synergic or antagonistic interactions in biofilms are of utmost relevance in maintaining health or altering the stability of the resident oral microbiome [39]. In the context of this complex network of inter-kingdom interaction has been enlightened a central role of *Candida albicans*, one of the most relevant fungal colonizers of the mouth. This commensal fungus entertains a multitude of synergic or antagonistic interactions with the oral bacterial microbiota that seems to influence oral bacterium behavior and promoting *C. albicans* survival in composite biofilms found on natural or prosthetic surfaces [40]. In this regard, *C. albicans* structures play a protective role for pathogenic bacteria such as *P. gingivalis* from the recognition by the host immune cells and it can support bacterial gingival infections [41].

Perturbation in the composition and function of the indigenous oral microbiome may determine an alteration of the symbiotic interaction between the oral microbial community and the host with consequences for the oral and general health of the individual. The alteration of this finely-tuned equilibrium between host and hosted microbes (dysbiosis), allows pathogenic bacteria to manifest their disease-promoting potential and determinate pathological conditions [7]. Our drastically increased understanding of the dynamic interactions between the various microbial and host factors has led to a new microbial model of periodontal pathogenesis, according to which the pathogenic process that drives periodontal tissue destruction is not related to a limited number of periodontopathogenic species but is the outcome of a synergic action of dysbiotic microbial communities [42]. For example, *P. gingivalis*, one of the major etiologic microbial agents of periodontitis included in the Socransky Red Complex, requires iron and protoporphyrin IX from heme to survive and support dysbiosis initiation and development and the consequent onset of chronic periodontal disease [43]. Dysbiosis seems to be globally associated with an increase in microbial diversity since the perturbation of the microbial environment allows certain indigenous species to expand and provides ideal conditions for the growth advantages of opportunistic microbes [44]. Several opportunistic pathogens were frequently detected in the periodontal microbiota, including oral commensal microbes—like *Neisseria* spp. or *E. saphenum*—and no oral colonizers species that may disseminate to other areas of the body and potentially lead to the development of infections of soft tissues, abdominopelvic cavity, and endocarditis, especially in immunodeficient and traumatized individuals [45]. However, changes in microbial diversity between health (eubiosis) and periodontal disease (dysbiosis) remain controversial, since some researchers reported a loss of microbial diversity, other indicated an increasing level of microbial diversity and still others did not report significant differences [46].

**Figure 2:**
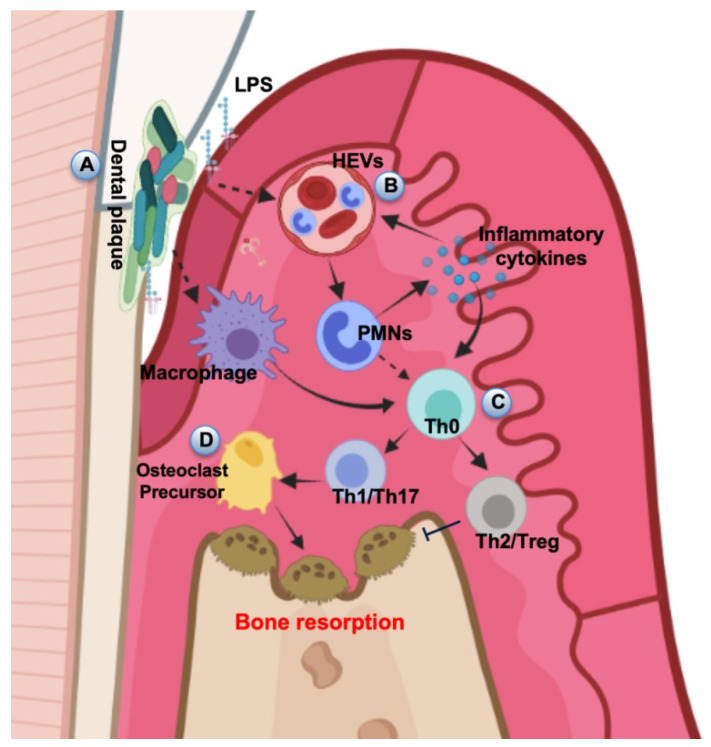
A diagram by Jiang et al. [49]. (**A**) In periodontal tissue, the dental plaque stimulates local inflammatory and immune responses. (**B**) LPS and other plaque PAMPs as well as DAMPs activate the HEVs leading to vascular hyperpermeability and leakage PMN transmigration. (**C**) APCs interact with naive T helper cells, driving their differentiation into several subsets. (**D**) The amplification of local immune response leads to the development of inflammation and results in the progression of periodontal destruction and bone resorption. Under the terms of the Creative Commons Attribution License (CC BY).

The host immune-inflammatory response in periodontitis (Figure 2) is initially characterized by a physiological acute inflammation reaction (gingivitis) to supragingival and subgingival plaque, sustained by the cell of the innate immune system, including resident cells (epithelial cell and fibroblast), phagocytic cells (macrophages and neutrophils), complement proteins and neuropeptides. In this phase, cytokines produced by the residential cell population such as tumor necrosis factor (TNF)-α, interleukin (IL)-1β, and interleukin (IL)-6 have the main function to stimulate cells migration to sites of infection and enhance the expression of adhesion molecules for neutrophils on the internal vessel surfaces and increase the synthesis of other proinflammatory cytokines [47]. The elimination of plaque leads to the progressive resolution of the inflammation and the restoration of individual homeostasis. Plaque persistence results in the activation of acquired immunity. This event occurs through antigen processing and presentation by lymphocytes, macrophages, and dendritic cells and is regulated by adaptive-immunity cytokine, including interferon (IFN)-γ and interleukin (IL)-2 and interleukin (IL)-4 [48]. The progressive destruction of periodontal tissues leads to the resorption of bone tissues and the degradation of ECM. Bone resorption results from the shifting balance between osteogenesis and osteoclastogenesis in favor of the second one, which is governed by a complex inflammation-induced osteoclastogenesis pathway, involving the receptor RANK and its ligand (RANKL), IL-1B, IL-6, and TNF-α [47]. The degradation of ECM is due to the up-regulation of the expression of a family of 23 Zn^2+^- and Ca^2+^-dependent enzymes, known as matrix metalloproteinases (MMPs). These enzymes are normally characterized by a low expression level in healthy periodontal tissues, where are involved in some physiological functions, like tissues development and turnover. MMPs also contribute to pathological processes such as the degradation of gingival and periodontal ligament collagen that occurs in periodontitis during connective tissue catabolism [48]. Moreover, Nitric oxide (NO) has been considered as a biological marker of oral bacterial pathologic activity in oral diseases. More specifically, it has been showed that salivary nitric oxide (NO) levels are more elevated in patients with periodontal disease compared to healthy individuals, indicating that NO levels correlated to worsened periodontal parameters were the result of the bacterial induced inflammatory response [49].

## 4. Impact of Environment and Periodontal Treatment on the Oral Microbiome

Environmental factors may considerably impact the oral microbiome composition and lead to the onset of lifestyle-related disorders. Accumulating evidence suggests that *P. gingivalis* increases the risk of metabolic, inflammatory, and autoimmune disorders through a mechanism that has not yet been elucidated [50].

Obesity, one of the most common lifestyle-related disorders, is associated with an increase of periodontal pathogens: a study carried out by Maciel et al. has shown that obese people with chronic periodontitis present an increased proportion of periodontal pathogens bacterial species, such as *Eubacterium nodatum*, *Aggregatibacter actinomycetemcomitans* and *Fusobacterium nucleatum*, compared to those with normal weight and affected by chronic periodontitis [51]. Numerous studies have revealed that smoking represents one of the most relevant risk factors for the initiation and progression of periodontitis [52,53,54] and also an important predictive factor for the success of non-surgical and surgical periodontal therapy [55]. Tobacco smoking can directly promote changes in the microbial environment through direct contact with the members of the microbial community, or indirectly, by interfering with the host immune system, biofilm formation process, or oxygen tension [56]. Furthermore, although the mechanism by which smoking contributes to the destruction of periodontal tissues has not been identified, smoking action seems to induce an impairment of neutrophilic granulocytes chemotaxis and phagocytic function, to interfere with humoral immunity impairing Ig-G and Ig-E serum levels, and to increase the synthesis of reactive oxygen species (ROS) and the release of some proteases, like collagenase and elastase, as well as proinflammatory cytokines, in periodontal tissues [57]. A diet low in vitamins and other micronutrients is related to major periodontal disease severity; a positive and significant association between micronutrients intake and periodontal disease severity was found for vitamin A, B1, C, and E, iron, folate, and phosphorus [58,59,60].

Restorative dental materials and dental prostheses, due to their specific physical and chemical characteristics, can influence biofilm formation and development. Rough surfaces compared to smooth ones provide more irregular regions that are ideal for microbial colonization and promote plaque formation and maturation. Amalgam restorations surfaces show a barely viable biofilm if compared to other materials probably as a result of the release of bacteriostatic and bactericidal ions from amalgam, such as Hg, Ag, and Zn [61]. In vivo, ceramic materials differed by their capacity of promoting biofilm accumulation in relation to their composition and microstructure; in particular, zirconia seems to under favor biofilm accumulation compared to other ceramic materials [61]. Resins-based composites are variably prone to resin biodegradation depending on changeable proportions and distribution of resin matrix and filler particles on the surface of those materials. The biodegradation products derived from residual monomer release from composites enhance biofilm growth in vitro, but further studies are required to verify if this action is less pronounced in vivo, considering that the dynamic changes that occur in the oral cavity are not reproducible with in vitro experimental protocols [61]. Glass ionomer cements, thanks to their fluoride-releasing, seems to influence acid production and adaption in biofilm dental plaque—especially during the early formation phases—and to variably affect biofilm growth according to many experimentally tested conditions, such as sterile environment or pH value [62].

Periodontitis treatment aims to prevent future disease development, minimize symptoms and reduce the risk of tooth loss, perhaps restoring damaged periodontal tissues, and proving information to patients on how to maintain periodontal health (Table 1). The first step of periodontal therapy includes several educational interventions like dental hygiene instructions, smoking quitting programs, dietary and other lifestyle modifications, aiming at improving the awareness and the adherence of the patients to treatment and in the post-treatment period over time [63]. The second step of periodontal therapy known as cause-related therapy, involves supragingival instrumentation with professional mechanical plaque and calculus removal (PMPR), in association with the control of retentive plaque factors, essential to reducing deep pocket inflammation and improving clinical attachment levels (CAL). Subgingival instrumentation may be performed with different approaches and can be associated with the use of adjunctive chemical agents and local and systemic antimicrobial agents [63]. Quadrant-wise debridement scaling and root planing (Q-SRP) represents the conventional methodic and consists in scaling and root planing (SRP) of each mouth quadrant in separate sessions, each one separated by a minimum interval of one week. Full-mouth scaling (FMS) and Full-mouth disinfection (FMD) are alternative approaches consisting of SRP of all mouth quadrants in 24 h; in addition, the second modality involves the adjunctive use of an antiseptic agent, such as chlorhexidine (CHX) [64]. The adjunctive subgingival administration of CHX to nonsurgical periodontal treatment (NSPT) seems to provide a probing pocket depth (PPD) reduction in patients with chronic periodontitis compared to those treated with NSPT without additional chemical therapy [65]. Interestingly, patients with generalized chronic gingivitis had more alkaline salivary PH and patients with generalized chronic periodontitis had more acidic pH compared to healthy individuals, showing that PH alteration may depend on the severity of the periodontal inflammation [66]. In this regard chlorhexidine mouthwashes induce a lowering of salivary pH involving increased risk of tooth demineralization. Therefore, in terms of salivary pH, CHX chlorhexidine could be more useful in the management of gingivitis than periodontitis [67]. Although chlorhexidine is one of the most effective antimicrobial agents, it is not without side effects. The main reported adverse effects caused by chlorhexidine mouthwashes include parotid gland swelling, pigmentation of the oral soft tissues and teeth, type 1 hypersensitivity reactions, taste alteration, burning sensation, oral mucosa ulceration or erosions, a transient anesthetic sensation, and paresthesia [68]. Therefore, consideration should be given to when the use of this antimicrobial agent is most indicated in the management of gingival and periodontal conditions.

Enough evidence suggests that the adjunction of systemic antimicrobials to scaling and root planing (SRP) improves the clinical outcomes in the mechanical treatment of chronic and aggressive periodontitis, not only in terms of CAL gain and PPD reduction [69,70] but also in terms of reduction of risk of additional CAL loss; especially SRP combined with spiramycin or amoxicillin/metronidazole, showed respectively significant PPD and CAL modifications in deep pockets [71]. The administration of systemic antibiotics during NSPT should always be judicious and restricted to specific periodontal patients—such as those affected by aggressive or progressing periodontitis—[72] and cannot be maintained any longer, considering the systemic side effects and the microbiological adverse effects as well as the significant risk of development and increasing of antimicrobial resistance [73]. Local delivery of statins, such as simvastatin, as an adjunct to SRP, seems to improve clinical benefits, including CAL gain, PPD reduction, and minor development of intrabony (IB) defects, even in smokers and diabetic patients [74,75]. The adjunctive use of essential oil-based mouthwash (EOBM) to SRPA has shown clinical outcomes, in a short-term follow-up, that are consistent with those reported for CHX [76] and has proven to improve the effectiveness of periodontal treatment, as compared to SRPA is performed alone, in different types of chronic periodontal patients, including type-2 diabetic (T2D) [77], smokers [78] and rheumatoid arthritis [79].

Almost all methods of mechanical periodontal treatment seem to benefit from supplementary antimicrobial chemotherapy, and no one treatment option is significantly superior for treating chronic periodontal disease independently from the periodontal biotype [80,81].

Schwarzberg et al. studied the differences in bacterial composition in healthy and periodontal sites after standard periodontal treatment using NGS methods. The analysis revealed that post-treatment samples were remarkably similar to pre-treatment samples from the same individual with notable changes in the amount of *Fusobacterium* and *Prevotella* species [82]. 6-month research evaluating the subgingival microbial longitudinal outcome of different non-surgical treatment approaches—quadrant-wise debridement scaling and root planing (Q-SRP), full-mouth scaling (FMS), full-mouth disinfection (FMD), and FMD combined with the use of erythritol air-polishing (FMDAP)—performed in patients with stage III and IV periodontitis, show a drop in pathogenic bacteria 3 months after the treatment, which was followed by a resurgence 6 months later [83].

Subgingival instrumentation can be re-performed during the third step of periodontal treatment, in presence of periodontal sites that failed to respond properly to the second step of treatment; in presence of intrabony defects, furcation lesions, and persistent residual pockets, the treatment is directed to surgical intervention with access flap, resective or regenerative approaches [63].

## 5. Oral Probiotics, Prebiotics, and Symbiotic in the Treatment of Dysbiosis Induced by Periodontitis

Probiotics are living microorganisms naturally contained in certain foods or dietary supplements that, when administered in controlled doses, can confer several benefits to individual health [84] including microbial balance modulation, the enhancement of the immunity system, the antihypercholesterolemic and antihypertensive action, and the reduction of diarrhea associated with irritable bowel syndrome [85]. The positive impact of probiotics consumption on gastrointestinal health is well documented [86] and their effectiveness against naturally emerging microbiome imbalance accounting in other human body sites, like the oral cavity, has recently been investigated over the last decade. According to several human clinical trials, probiotics have the potential to modify the composition of the sub-gingival microbiota, lowering considerably the concentration of the major periodontal pathogens, and can be used as adjuvant agents to reinforce the clinical improvements provided by mechanical debridement, all without any kind of evidence of short- and long-term side effects in the patients [87,88,89]. Many lactobacilli and streptococcal strains have been shown to have antibacterial activity against periodontopathic bacteria such as *P. gingivalis*, *S. mutans*, *P. intermedia*, and *A. actinomycetemcomitans* in in vitro investigations [90,91]. Lactobacilli antibacterial activity can also affect the host immunological reactivity, regulating the periodontal pathogen-induced inflammatory response. This modulating action on the host immunity system was experimentally observed in a co-culture between *L. acidophilus*, *P. gingivalis,* and human gingival epithelium cells, where the expression of the inflammatory cytokines IL-1B, IL-6, and IL-8, induced in gingival epithelial cells by *P. gingivalis* infection, was reduced in presence of an increasing concentration of *L. acidophilus* [92]. An investigation carried out on a murine model of periodontitis (ligature-induced periodontitis) revealed that *L. brevis* CD2 bacteria canreduce periodontal inflammation and bone loss as well as the number of anaerobic bacteria, which are significantly related to the illness [93].

Prebiotics are additional unviable substances promoting selectively the growth and/or the activity of resident microorganisms associated with host health, including also some probiotic species such as *Lactobacillus* and *Bifidobacterium* [94]. According to the actual evidence, are considered prebiotics some oligosaccharides like fructooligosaccharides (FOS), galactooligosaccharides (GOS), mannanoligosaccharide (MOS), andxylooligosaccharide (XOS), Human milk oligosaccharides (HMOs) and inulin; some fatty acids, including conjugated linoleic acid (CLA) and, polyunsaturated fatty acid (PUFA). Furthermore, certain substances act as prebiotic deadening on the target site; for instance, XOS is currently considered a prebiotic substrate only in the oral cavity [94]. Several studies indicate prebiotics have a beneficial effect on the immune system of the host, enhancing immune functions directly, through the up-regulation of anti-inflammatory cytokines and the down-regulation of pro-inflammatory cytokines, and indirectly, modifying the composition and activities of gastrointestinal microbiota [95]. Prebiotics represent a recent introduction in the field of oral health. There is evidence that in vitro N-acetyl-D-mannosamine stimulates the growth of beneficial bacteria, like *S. sanguinis*, *S. mitis,* and *S. oralis*, in biofilm with multi-species composition, especially in nutrient-rich environments, with a dose-dependent effect. However, studies in vivo are required to confirm the potential role of this compound as a prebiotic substance [96].

Symbiotic agents are a mixture of living organisms and specific substrates, which can be selectively utilized by the co-administered live microorganisms (synergistic symbiotic agents) or by the resident or colonizing microflora of the host (complementary symbiotic agents in which the living microorganisms and the substrates meet the criteria of probiotic and prebiotic respectively) [97]. Beneficial effects provided by symbiotic agents on individual health are today subjects of great attention in scientific research. Weak evidence suggests that the oral administration of symbiotic in association with probiotics could help prevent and treat some dysmetabolic disorders like obesity, T2D, insulin resistance syndrome (IRS), and non-alcoholic fatty liver disease (NAFLD) [98]. The outcomes of a double-blind randomized controlled trial show that symbiotic are capable to reduce significantly the level of *S. mutans* in saliva children after 15 days of daily intake, but seem to be less effective in inhibiting the growth of *S. mutans* compared to salivary samples of children who have taken probiotics daily for the same period [99].

## 6. Future Challenges

Currently, the therapy of choice in the treatment of periodontitis is the mechanical removal of dental plaque above and below the gum line, which may or may not be accompanied by the use of adjuvants, such as antiseptics and antimicrobials. These substances induce only momentary effects and cannot be used in long-term therapy. Therefore, current therapeutic strategies induce only a momentary modification of the oral microbiota, which explains the temporary improvement of clinical periodontal parameters following scaling and root planning. Although further studies in this field are needed, a proper lifestyle and use of agents able to promote restoration of oral eubiosis and prevent oral dysbiosis (i.e., probiotics and prebiotics), represent a true revolution in the prevention and clinical management of periodontal disease in the long term and also a valuable therapeutic aid when integrated with Q-SRP and FMS mechanical plaque removal approaches.

## 7. Conclusions

Periodontitis is a chronic and extremely widespread diseases that affects irreversibly the tooth-supporting tissues comporting irreparable damages to oral health. The role of the oral microbial community in maintaining an oral and systemic health status is increasingly clear as well as modern lifestyle, including an unbalanced diet and smoking, along with bad oral hygiene, represent the main factors promoting the upset of the harmonious equilibrium of the oral microbiome and the onset of oral disease like periodontitis, especially in individuals with genetic and epigenetic susceptibility. In this perspective, educating patients on a a culture of oral prevention and healthy lifestyle choices and using efficient plaque treatment strategies that maintain the natural diversity of resident microbiota should represent the main goals in the prevention and treatment of periodontal disease.

## Figures and Tables

**Table 1:** This table summarizes the main characteristics of the stepwise approach-based periodontal treatment according to the available evidence in the EFP S3 level clinical practice guideline [62].

Steps of Therapy	Interventions	Endpoints of Therapy
**First Step Therapy** in all periodontal patients	Oral hygiene instructions (OHI) and other educational interventions to improve patient motivation and adherence	Build motivation and adherence of periodontal patients to horal hygiene and to obtain behavior changes useful to take under control periodontitis- related risk factors
	Supragingival dental biofilm control	
	Professional mechanical plaque removal (PMPR)	
	Risk factor control (smoking cessation, metabolic control of diabetes, dietary counselling and weight loss and improved physical exercise)	
**Second Step Therapy** in teeth with loss of periodontal support and/or periodontal pocket formation	Subgingival instrumentation with or without adjunctive therapies (physical or chemical agents, local or systemic host-modulating agents, local or systemic antimicrobials)	Absence of periodontal pockets >4 mm with bleeding on probing or deep periodontal pockets ≥6 mm
**Third Step Therapy** in those sites with the presence of pockets ≥4 mm with bleeding on probing or presence of deep periodontal pockets ≥6 mm	Repeated subgingival instrumentation with or without adjunctive therapies. Surgical intervention (access flap periodontal surgery, resective periodontal surgery, regenerative periodontal surgery) to gain further access to subgingival instrumentation or to treat periodontal lesions associated with intra-bony defects and furcation involvement	Obtain periodontal stability and place patient in Supporting Periodontal Care (SPC) program

## Data Availability

Data are available from the Corresponding author upon reasonable request.
